# Hydride Shuttle Catalysis: From Conventional to Inverse Mode

**DOI:** 10.1021/jacsau.4c00532

**Published:** 2024-08-19

**Authors:** Iakovos Saridakis, Immo Klose, Benjamin T. Jones, Nuno Maulide

**Affiliations:** † Institute of Organic Chemistry, University of Vienna, 1090 Vienna, Austria; ‡ Vienna Doctoral School in Chemistry (DoSChem), University of Vienna, 1090 Vienna, Austria

**Keywords:** hydride shuttle catalysis, borane catalysis, frustrated Lewis pairs

## Abstract

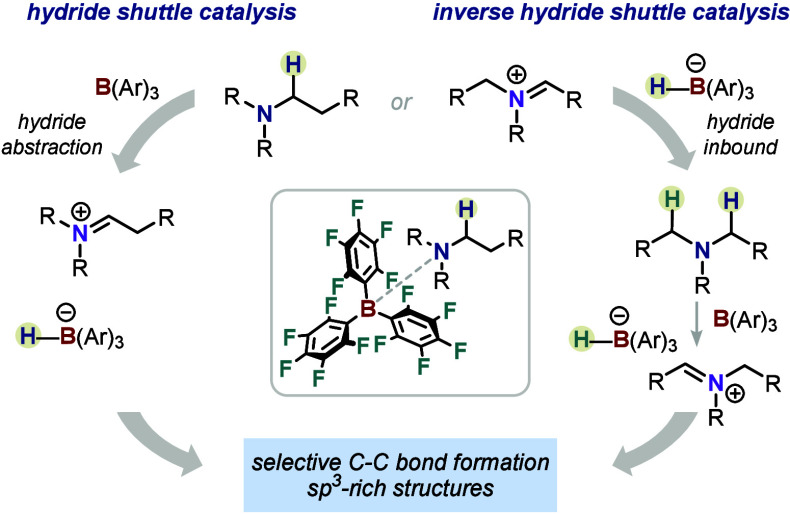

Hydride shuttle catalysis has emerged as a powerful synthetic platform, enabling the selective formation of C–C bonds to yield sp^3^-rich structures. By virtue of the compelling reactivity of sterically encumbered Lewis acids from the frustrated Lewis pair regime, hydride shuttle catalysis enables the regioselective functionalization of alkyl amines at either the α- or β-position. In contrast to classical Lewis acid reactivity, the increased steric hindrance prevents interaction with the Lewis basic amine itself, instead leading to reversible abstraction of a hydride from the amine α-carbon. The created positive charge facilitates the occurrence of transformations before hydride rebound or a similar capture event happen. In this Perspective, we outline a broad selection of transformations featuring hydride shuttle catalysis, as well as the recently developed approach of *inverse* hydride shuttle catalysis. Both strategies give rise to a wide array of functionalized amines and offer elegant approaches to otherwise elusive bond formations.

The reaction of a Lewis acid with a Lewis base, leading to the formation of a dative covalent bond, was first described a century ago by Gilbert Lewis and constitutes textbook knowledge.^1^ However, when steric hindrance or geometrical constraints hamper the approach of the two species, a classical acid–base adduct is not formed. Instead, the unquenched reactivity of both species leads to a chemical behavior entirely distinct from that of the individual components. Such reactivity was first described in 1942 by H. C. Brown, who found the sterically encumbered Lewis base 2,6-lutidine to be unable to form a dative covalent bond with trimethylborane.^2^ However, it was not until 2006 that the potential of this phenomenon was realized by a report by Douglas Stephan on H_2_ splitting using a unimolecular phosphine-borane pair. This marked a breakthrough in the field, which was has since been referred to frustrated Lewis pair (FLP) chemistry.^3−7^ Ever since, FLPs have pioneered transformations such as metal-free catalytic hydrogenation, the capture of other small molecules (*e.g.*, CO, CO_2_, or SO_2_) and, more recently, C–H borylation and silylation.^8^

Shuttle catalysis has emerged over the past several years as a powerful technology for the construction of valuable motifs, often circumventing the use of toxic or highly reactive reagents.^9,10^ In the shuttle catalysis platform, a functional group (*i.e.*, the shuttled group) is formally transferred from a donor species to an acceptor, thus achieving selective functionalization of the acceptor (or, conversely, defunctionalization of the donor) (Figure 1a). The most extensively studied groups within the field of shuttle catalysis include hydrogen (transfer hydrogenation), syngas (H_2_/CO), water, reactive species such as carbenes, and reagents such as HCN.^11^

**Figure 1:**
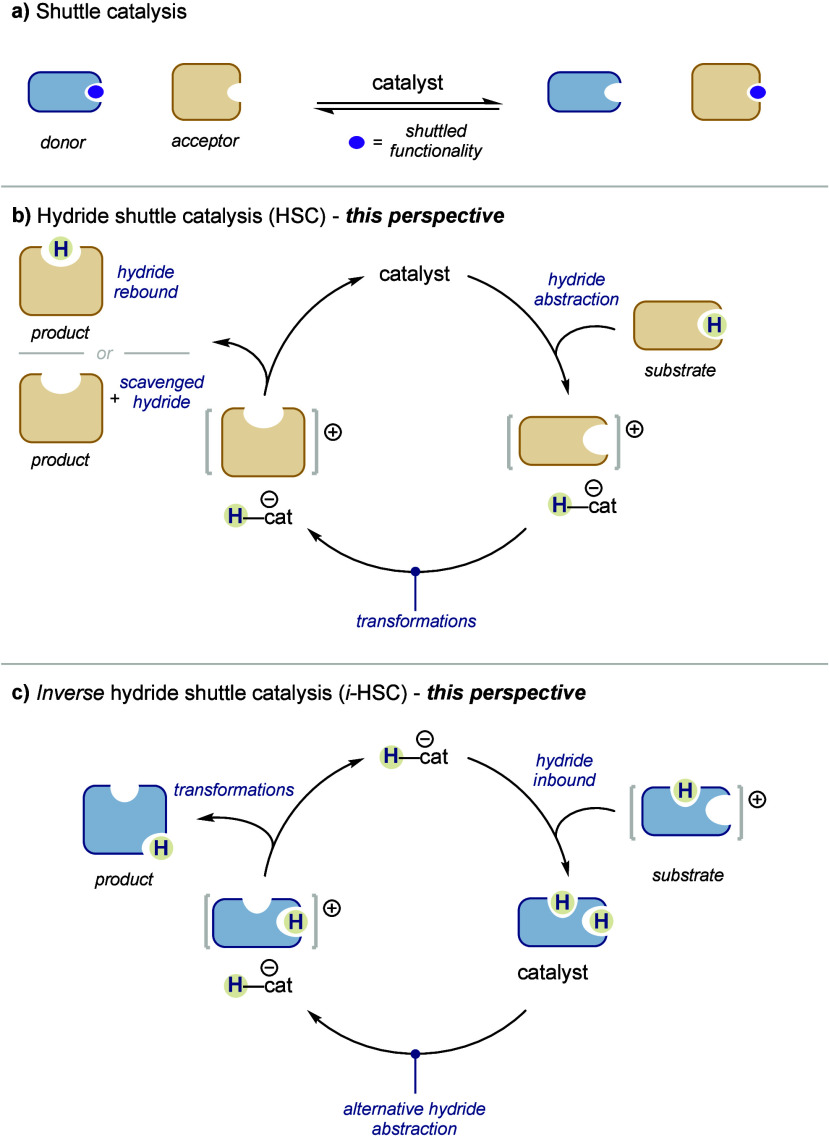
General concepts of (a) shuttle catalysis, (b) hydride shuttle catalysis, and (c) inverse hydride shuttle catalysis.

More recently, *hydride shuttle catalysis* (HSC) has been described as a unique catalysis platform with synthetic potential, closely associated with the unique functions and properties of FLPs. In brief, a borane Lewis acid (*e.g.*, B(C_6_F_5_)_3_) captures a hydride from a starting material capable of acting as a hydride donor (*e.g.*, the α-hydrogen of an alkylamine), generating an ion pair (*e.g.*, borohydride and iminium ion).^12−15^ Rearrangements or other transformations of the cationic intermediate are then followed by rebound of H^–^ from the borohydride, completing the hydride shuttle cycle and releasing the products (Figure 1b, hydride rebound). *As opposed to shuttle catalysis, which focuses solely on the transposition of functionalities, the distinct feature of hydride shuttle catalysis is the (often reversible) hydride abstraction/donation which unlocks otherwise elusive reactivity.* Closely related reactions have also been reported, in which the abstracted hydride does not end up incorporated within the product. Instead, the hydride is transferred to a scavenger (*e.g.*, trityl cation) or a sacrificial equivalent of substrate. Although these examples do not strictly fall into the above definition of hydride shuttle catalysis, they are closely related and provide important context for the current state of the art in this field. For this reason, these examples are also discussed in this perspective (Figure 1b, scavenged hydride products).

An alternative scenario is invoked by what is termed inverse hydride shuttle catalysis (*i*-HSC): a reaction platform with a series of transformations commencing with donation, rather than abstraction, of a hydride from the catalyst to the substrate (Figure 1c). This initially required hydride species can either be preformed or generated *in situ* through abstraction of hydride from the α-position of an amine.

In this Perspective, we highlight a selection of findings within the realms of HSC and the more recently developed *i*-HSC, ultimately discussing the selective functionalization of alkylamines and the formation of new C–C bonds using these approaches.

The first section of this article comprises HSC transformations involving addition of nucleophiles to transient iminium ions formed after hydride abstraction (Figure 2). The alternative pathway for HSC processes of alkylamines, discussed in the second section of this Perspective, involves α-deprotonation of the iminium ion by an appropriate base, forming a nucleophilic enamine ripe to engage in further reactions, such as electrophilic addition. In the last section of this article, we will discuss *i*-HSC, a comparatively novel technology.

**Figure 2:**
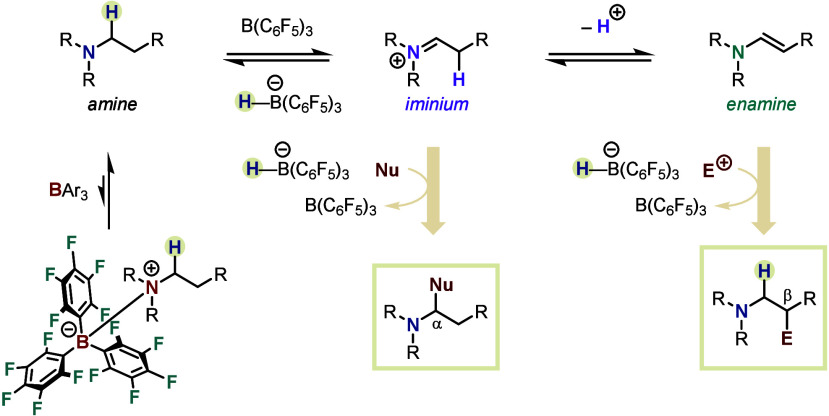
FLP enables HSC for the α- and β-functionalization of *N*-alkylamines.

From a conceptual point of view, we shall not cover transformations where the initially abstracted hydride is extruded in the form of H_2_.^16,17^ This also includes transfer hydrogenation,^18,19^ dehydrogenation,^20,21^ and transfer hydrosilylation in which the hydride is abstracted from a silane reagent rather than the substrate.^22−24^ While the Lewis acids mentioned in this review are exclusively boron based—the most common class—other Lewis acids, such as aluminum and a range of transition metals, are also capable of forming frustrated Lewis pairs.^25,26^

## A Hydride Shuttle Catalysis: Transformations of the Iminium Intermediate

**Scheme 1:**
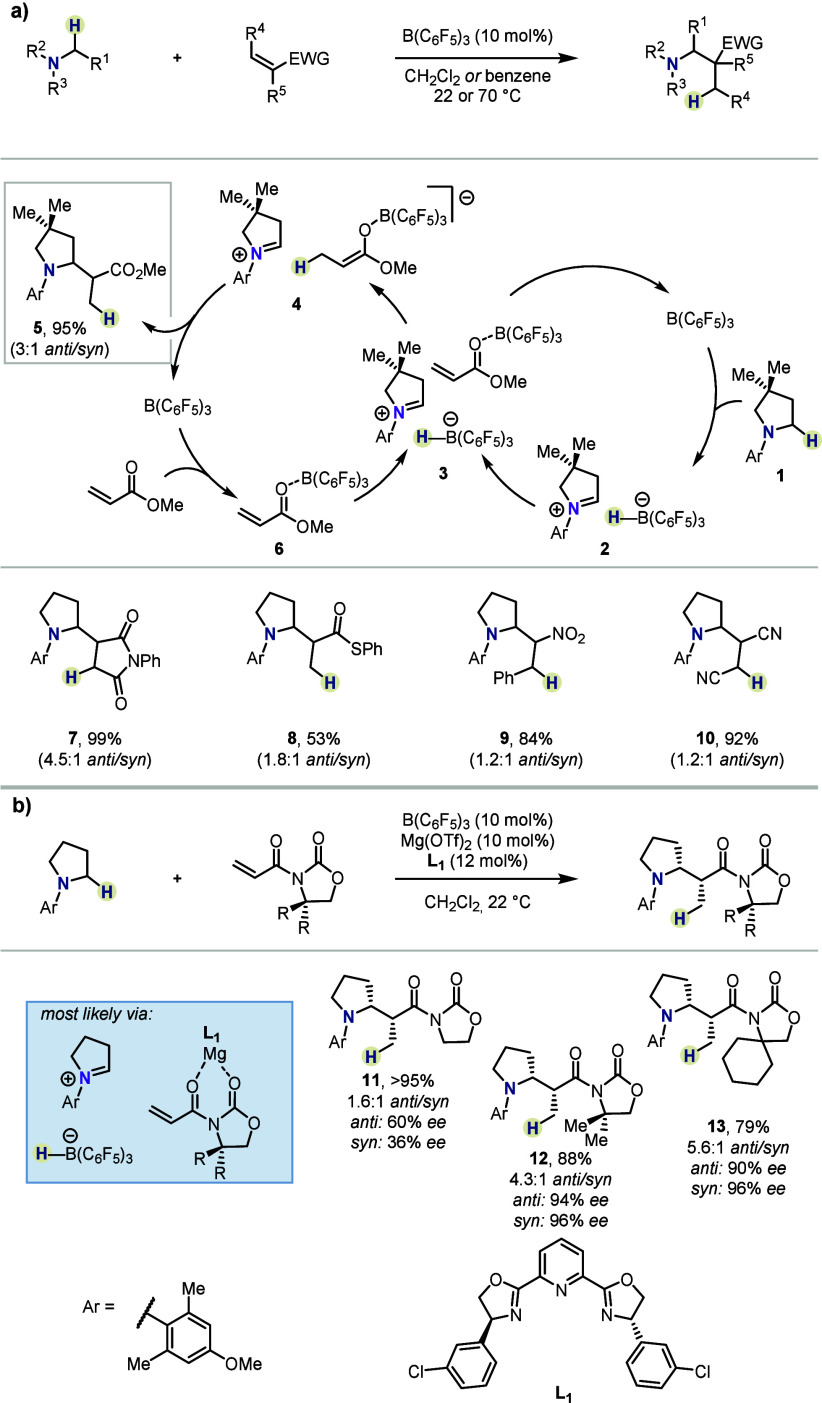
α-Functionalization of Amines by Wasa *et al.*

Early works in this area are based on the seminal work of Basset and co-workers in 2002 on borane-mediated hydride abstraction of amines,^27^ as well as work by Erker and co-workers on the α-functionalization of tertiary amines with stoichiometric amounts of B(C_6_F_5_)_3_.^28^ The first catalytic transformation of this type was investigated by the group of Wasa, who developed a stereoselective platform for the synthesis of Mannich-type products (Scheme 1a).^29^ Therein, alkylamines, *e.g.*, **1**, are oxidized by donating an α-hydride to B(C_6_F_5_)_3_ to form the corresponding iminium-borohydride ion pair **2**. Concurrently, the same Lewis acid catalyst activates an α,β-unsaturated carbonyl compound (**6**), setting the stage for 1,4-reduction by the borohydride, to generate a nucleophilic boron enolate species (**4**). Coupling of the latter with the previously formed iminium ion constructs a new C–C bond with moderate diastereoselectivity and releases the α-functionalized amine (**5**) with concurrent regeneration of the boron catalyst.

Remarkably, in the same work, Wasa and co-workers documented an enantioselective version of the developed reaction (Scheme 1b). Therein, boron catalysis, in conjunction with chiral magnesium Lewis acid catalysis, effectively couples α,β-unsaturated imides with alkylamines to deliver enantioenriched adducts with moderate to excellent levels of enantiomeric excess (*ee*). It must be highlighted that this approach offers synthetic advantages over the traditional Mannich reaction, where formation of enolates becomes challenging in the case of amides,^30^ thioesters, or other compounds with increased p*K*_a_ values of the α-protons.

Related intramolecular reactions have also been reported with the same Lewis acid by Huang and Paradies.^31,32^ However, in these cases an intramolecular hydride transfer is also conceivable. In fact, in the case of the work by Paradies *et al.*, mechanistic experiments and quantum chemical calculations suggest a mechanism consisting of a 1,7-hydride shift, rather than hydride shuttle catalysis, to be operative.

**Scheme 2:**
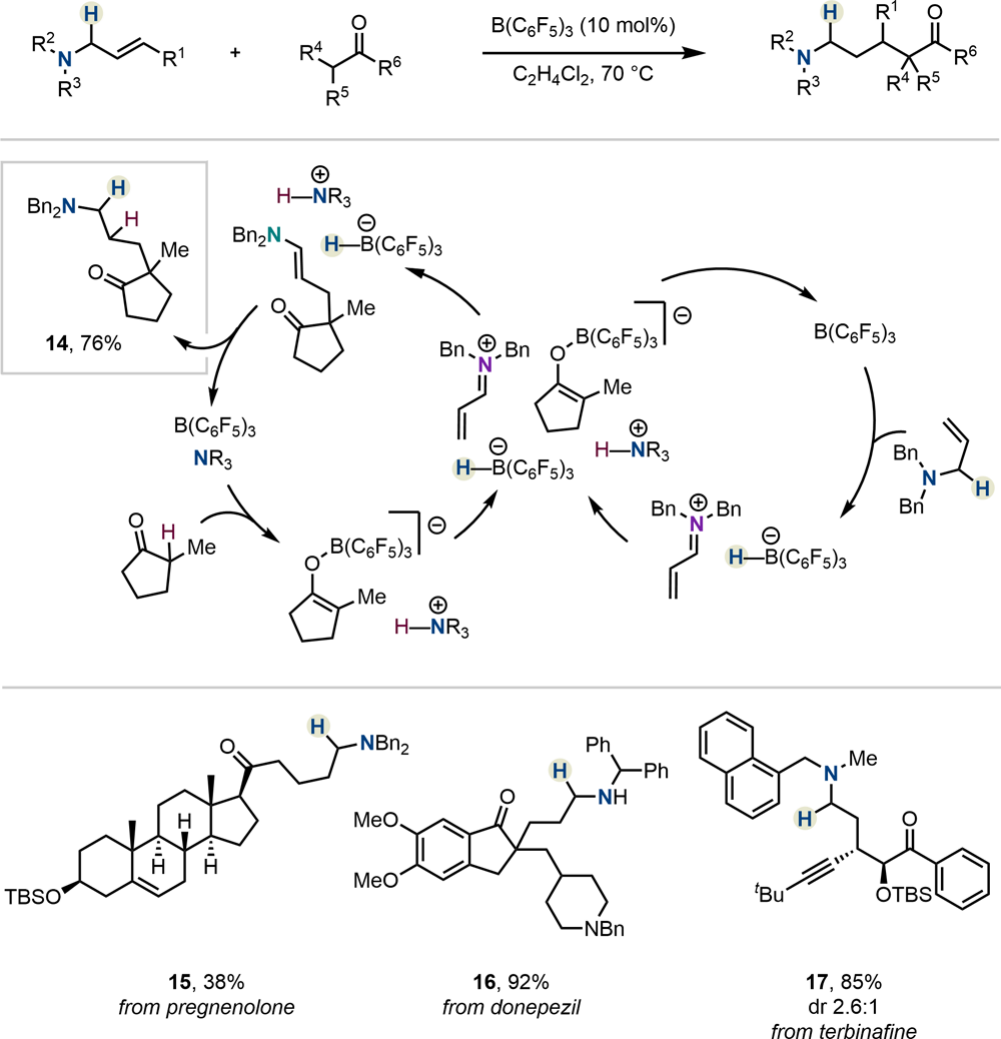
Synthesis of δ-Aminocarbonyls by Wasa *et al.*

Based on the above findings, Wasa and co-workers expanded the chemical space of amines accessible through HSC.^33^ In particular, incorporation of allylamines in a related reaction platform affords δ-aminated ketones (Scheme 2). The authors envisaged that the *in situ*-formed boron enolate—owing to its “soft” nucleophilic character—would preferentially perform a 1,4-addition at the unsaturated iminium ion. Indeed, a new C(sp^3^)–C(sp^3^) bond forms at the terminal position, simultaneously releasing an enamine. Protonation thereof, followed by rebound of the hydride from the catalyst, releases the new ketone (*cf*. **14**-**17**) or ester product and regenerates the catalyst. The protocol is robust, and the authors demonstrated its utility by late-stage modification of natural products and drugs or drug analogs (Scheme 2).

**Scheme 3:**
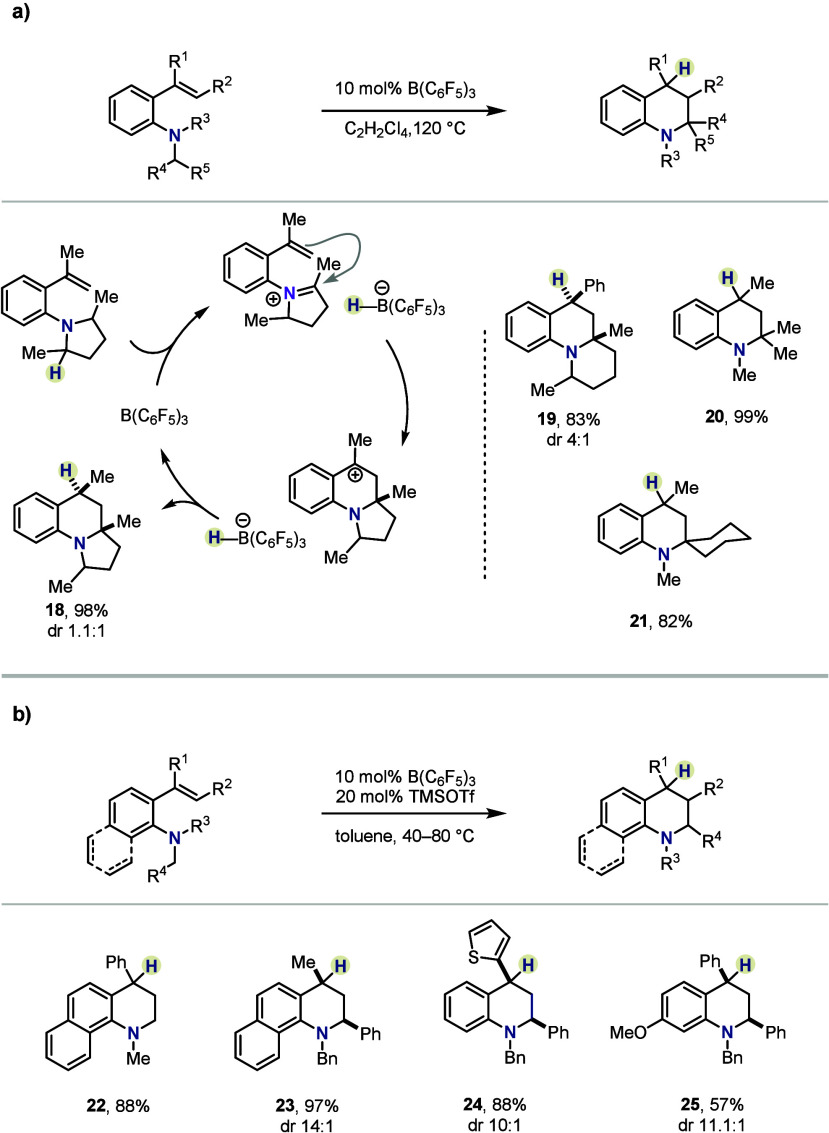
HSC-Enabled Synthesis of Tetrahydroquinolines by (a) Grimme, Paradies *et al.* and (b) Wang *et al.*

Applying a similar logic in 2018, Grimme and Paradies reported that B(C_6_F_5_)_3_ can catalyze the synthesis of tetrahydroquinolines from vinyl-substituted *N,N*-dialkyl anilines (Scheme 3a).^34^ There, B(C_6_F_5_)_3_ mediates a cyclization of the styrene double bond onto a transiently formed iminium ion, with the driving force being the formation of a stabilized benzylic, tertiary carbocation. The event is followed by neutralization of the positive charge through hydride rebound, furnishing the corresponding tetrahydroquinolines (e.g., **18**) and releasing the boron catalyst. The protocol allowed for the formation of a small yet diverse library of alkaloid scaffolds in moderate to excellent isolated yields, albeit with limited diastereoselectivity. Lastly, in some cases the authors reported β-elimination, preempting hydride rebound, as a side reaction, leading instead to dihydroquinoline products (not shown herein).

Independently, the research group of Wang reported almost concurrently a similar platform for the construction of tetrahydroquinolines from *o*-vinyl anilines in the presence of 10 mol % B(C_6_F_5_)_3_ (Scheme 3b).^35^ The method delivers a broad scope of alkaloids in moderate to excellent yields and with low to excellent diastereomeric ratios. Remarkably, the authors demonstrated that TMSOTf as a cocatalyst enabled formation of the desired quinolines at lower temperatures (40–80 °C), ascribed to the superiority of [TMS(OTf)H]^−^—the presence of which was proved experimentally—as a hydride donor.

The examples mentioned thus far involve rebound of the initially abstracted hydride onto transiently formed cationic intermediates, completing the catalytic cycle and liberating the borane catalyst. On the other hand, elegant examples exist that deviate from this route, and whereby closure of the catalytic cycle involves quenching of the hydride using a scavenger, thus enabling alternate synthetic avenues.

**Scheme 4:**
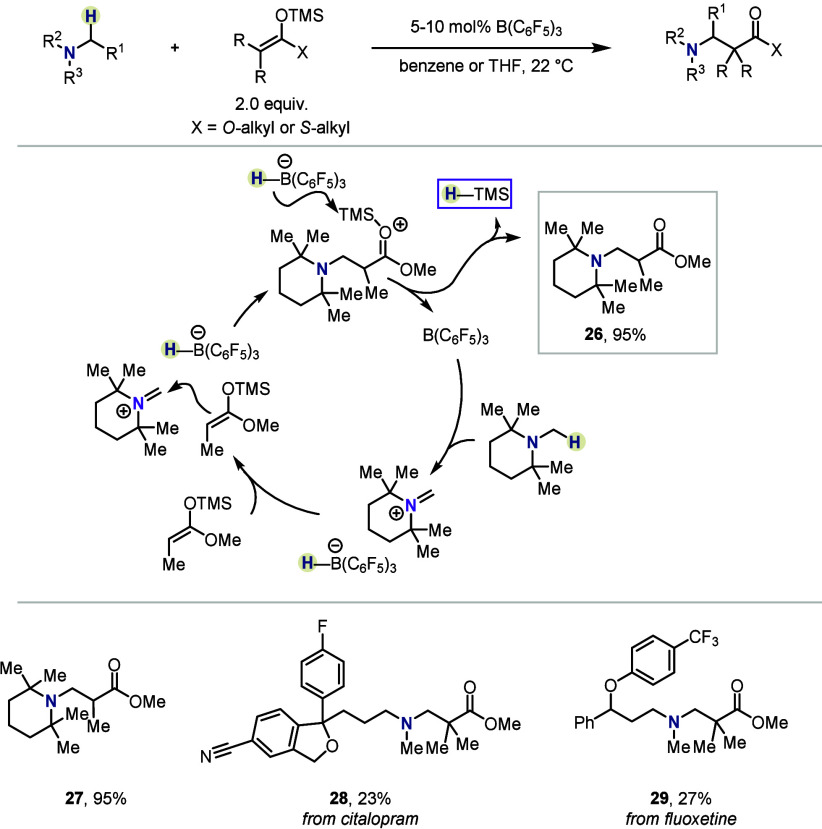
External-Oxidant-Free HSC-Enabled Synthesis of Mannich-Type Products by Wasa *et al.*

In 2019, the research group of Wasa expanded the portfolio of protocols for the borane-catalyzed construction of Mannich-type products (Scheme 4).^36^ In this report, silyl ketene (thio)acetals are selected as the nucleophilic reactants, engaging in nucleophilic additions to the iminium salts formed *in situ*. The formation of the new C–C bond produces a silylated carbonylonium ion, which—in the presence of [H–B(C_6_F_5_)_3_]^−^—readily releases the corresponding hydrosilane along with the desired amine. Contrary to the “conventional” oxidative Mannich addition, this protocol neither requires the presence of an organometallic catalyst, nor any stoichiometric oxidant additive. Both linear and cyclic tertiary amines were well tolerated, delivering the targeted β-aminocarbonyls in good to excellent yields. Late-stage functionalization of bioactive molecules, such as the antidepressant citalopram, demonstrated the applicability of the method to complex systems.

**Scheme 5:**
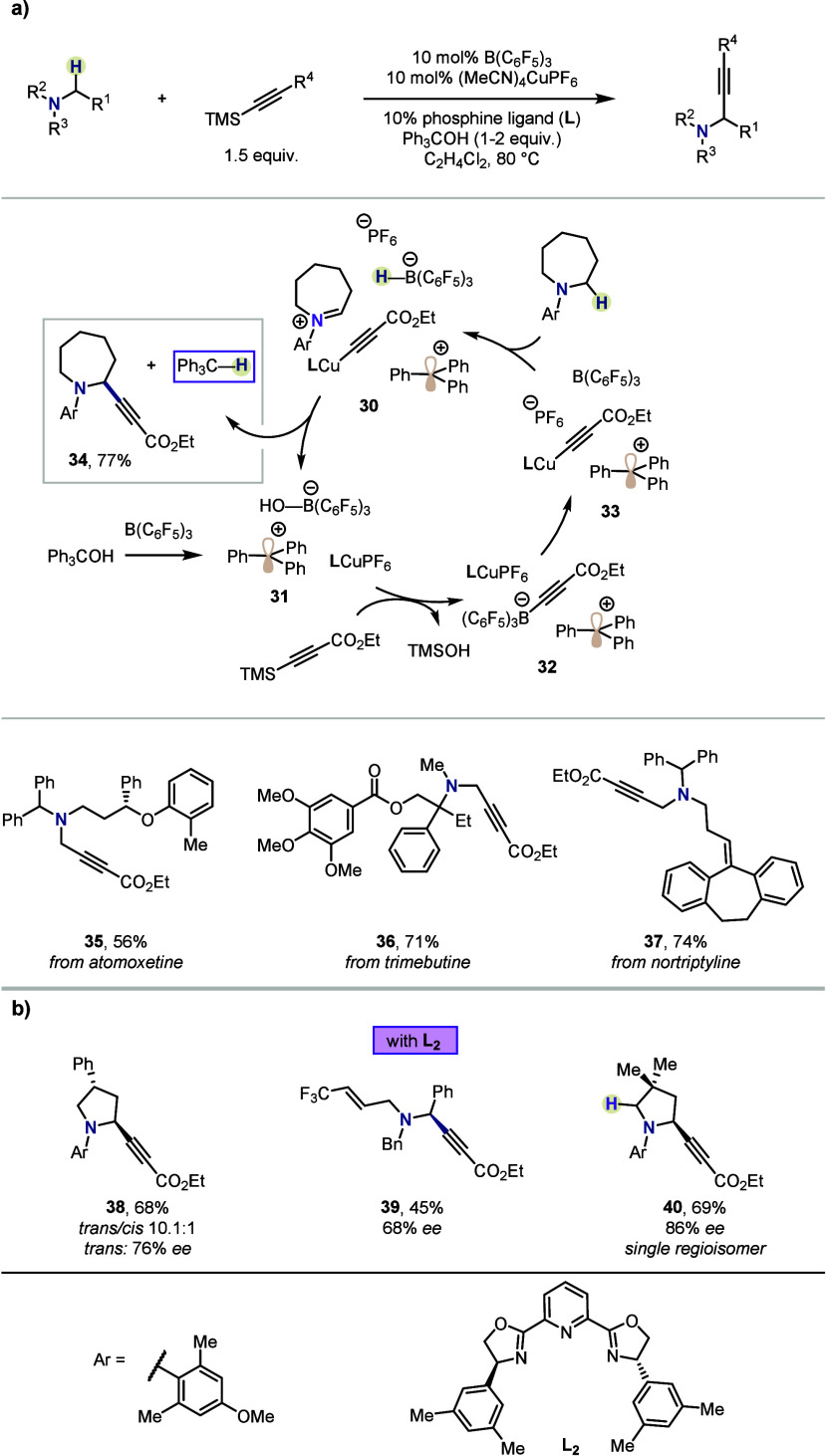
Synthesis of α-Alkynyl Amines by Wasa *et al.*

In 2020, the same research group established a new approach under the umbrella of hydride shuttle catalysis for the construction of C(sp^3^)–C(sp) bonds (Scheme 5a).^37^ In this report, cooperative catalysis employing borane and Cu(I) enables the coupling of alkylamines with alkynyl silanes, furnishing propargylamines, a motif found in various marketed pharmaceuticals (*e.g.*, selegiline). Although such couplings traditionally rely on stoichiometric oxidants (*e.g.*, O_2_),^38^ Wasa and co-workers envisioned that this requirement could be circumvented by the unique reactivity of FLPs. As such, the B(C_6_F_5_)_3_ Lewis acid catalyst was deployed with a double role: a) it readily abstracts a hydride from the alkylamine to form iminium (**30**) and b) is activated by a bulky alcohol (*i.e.*, Ph_3_COH), leading to a stable carbocation and borate [HO–B(C_6_F_5_)_3_]^−^ (**31**). The latter is believed to accelerate transmetalation onto a copper cocatalyst by virtue of the transient alkynyl borate [alkynyl–B(C_6_F_5_)_3_]^−^ (**32**), as supported by mechanistic investigations (including ^11^B NMR analysis). The resulting organocopper species (**33**) undergoes addition onto the iminium ion, irreversibly liberating the propargylamine product. Notably, in this case the abstracted hydride is not added back into the substrate, but instead the hydride is scavenged by the triphenylcarbenium ion. The protocol afforded a range of amines in moderate to excellent yields, and its robustness was highlighted through the site-selective modification of drug analogs. Notably, in the case of unsymmetrical amines, complete regioselectivity was observed for the least sterically encumbered C–H bond. This selectivity outcompeted even benzhydryl positions, which are inherently highly hydridic (*cf.***35**).

Moreover, the use of a chiral ligand (**L**_**2**_) allowed for enantioselective C(sp^3^)–C(sp) coupling (Scheme 5b). A series of enantioenriched cyclic or acyclic α-alkynyl amines were formed in up to 94% *ee*. In the case of the alkynyl pyrrolidine **38**, significant racemization was observed, most likely *via* reversible hydride abstraction of the more hindered site and enamine formation.

**Scheme 6:**
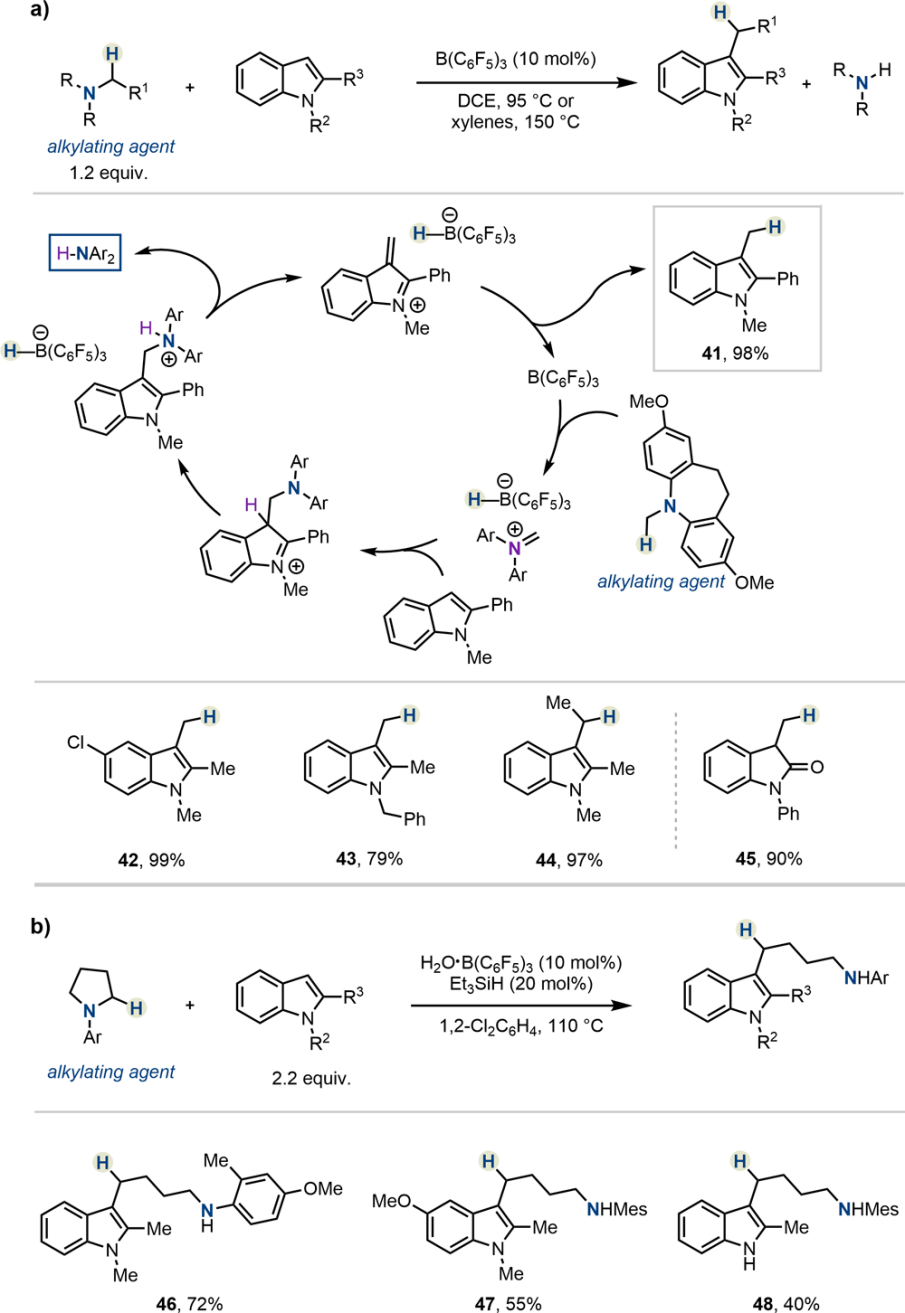
Alkylation of Indoles and Oxindoles with Amines by Melen, Morril, Pulis *et al.*

Complementing these studies, which helped conceptualize the field of hydride shuttle catalysis, in 2020 Pulis and co-workers reported a protocol for C-3 indole (or oxindole) alkylation in the presence of B(C_6_F_5_)_3_, using amines as the alkylating agents (Scheme 6a).^39^ More specifically, the *in situ* formed iminium ions are initially captured by the (ox)indole partner, followed by elimination of the (diaryl)amine to deliver a dearomatized, α,β-unsaturated iminium ion. The latter is quenched by hydride rebound, ultimately revealing the formally alkylated indole (or oxindole) and regenerating the borane Lewis acid catalyst.

Linear alkylamines were deployed to deliver a wide library of C-3-methylated, -ethylated, and -benzylated heterocycles, typically with excellent yields. Employing cyclic amines (such as N-arylated pyrrolidines), on the other hand, resulted in the formation of indole products carrying the deconstructed skeleton of the amine ring, following a cascade of alkylation/ring-opening (Scheme 6b). More recently, the authors expanded the scope of this transformation to α-secondary alkylamines (not shown herein).^40^

## B Hydride Shuttle Catalysis: Transformations at the Enamine Intermediate

**Scheme 7:**
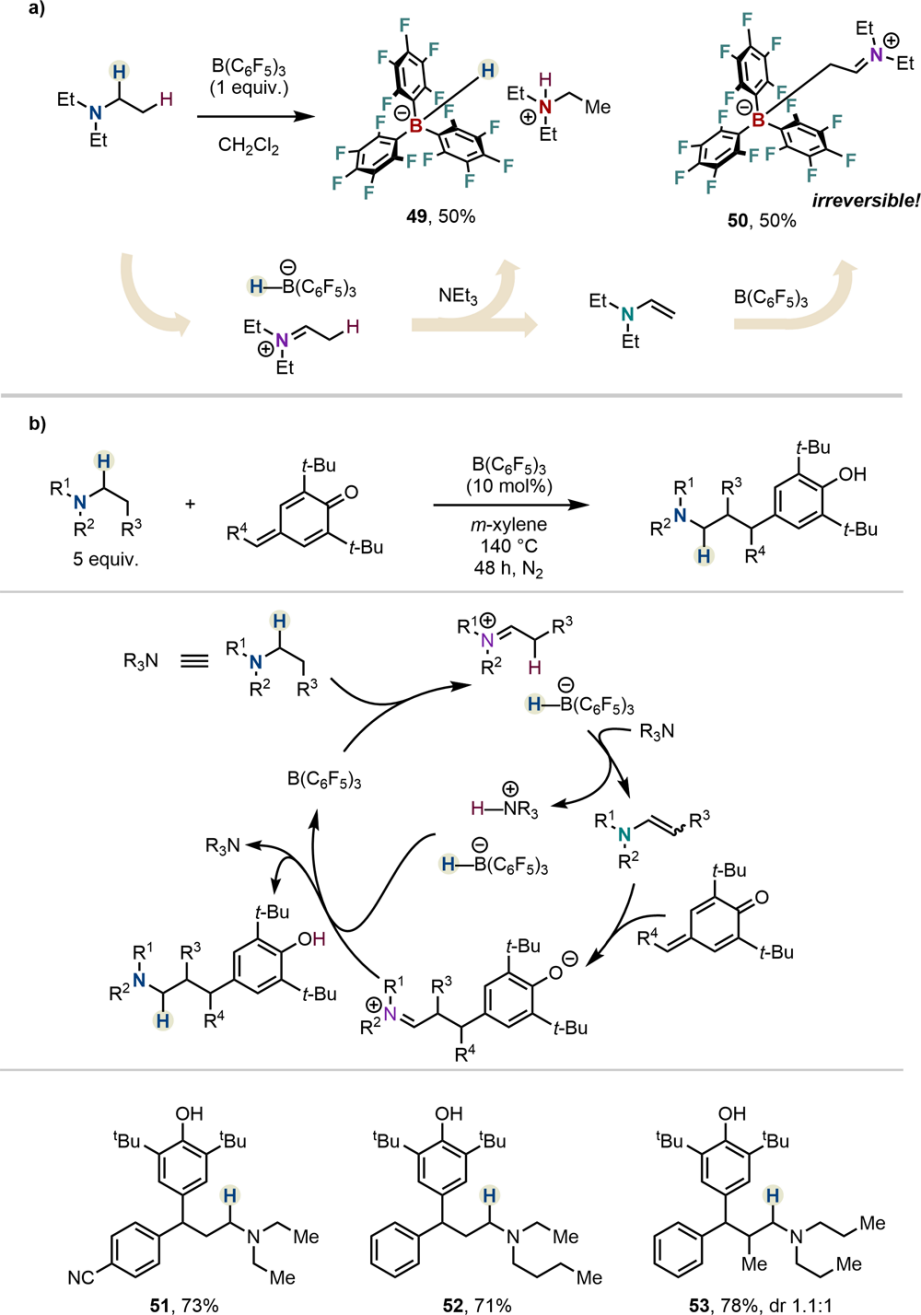
(a) Formation of Lewis Acid Adduct **50** and (b) β-Alkylation of Trialkylamines by Yang, Zhao, Ma *et al.*

For a period, enamine formation, as a result of α-deprotonation of the iminium ion after hydride abstraction, was seen as a considerable obstacle in this chemistry. Indeed, the enamine’s potential to react with, and potentially irreversibly quench, the Lewis acid catalyst by forming **50** (Scheme 7a) would shut down all possibilities of catalysis. Indeed, Resconi and co-workers demonstrated that enamines of nonsterically congested amines (*e.g.*, triethylamine) add irreversibly to B(C_6_F_5_)_3_), unless heating conditions are applied.^41,42^

Early catalytic examples feature aromatization of partially saturated nitrogen heterocycles to avoid Lewis adduct formation,^20,21^ with the groups of Kanai and Paradies demonstrating that the dehydrogenation was feasible under catalytic conditions. These reactions are termed “acceptorless” in comparison to examples in which the hydrogen is transferred to an acceptor molecule (for examples of dehydrogenation with acceptors, see Scheme 11 or Scheme 13).^43^

Yang, Zhao and Ma and co-workers disclosed the β-functionalization of amines using *p*-quinone methides as electrophiles (Scheme 7b).^44^ Therein, the enamines, formed through hydride abstraction and deprotonation, were found to add onto sterically hindered *p*-quinone methides featuring two α *t*-Bu substituents. Proton transfer and hydride donation completed the hydride shuttle catalytic cycle. Although the scope of the reaction remained limited, it demonstrated the ability of B(C_6_F_5_)_3_ catalysis to functionalize even unhindered trialkyl amines that suffer from Lewis acid adduct formation (see above). While adduct formation cannot be excluded, the high reaction temperatures likely allowed for its reversibility, thereby enabling irreversible C–C bond formation and catalytic turnover.

**Scheme 8:**
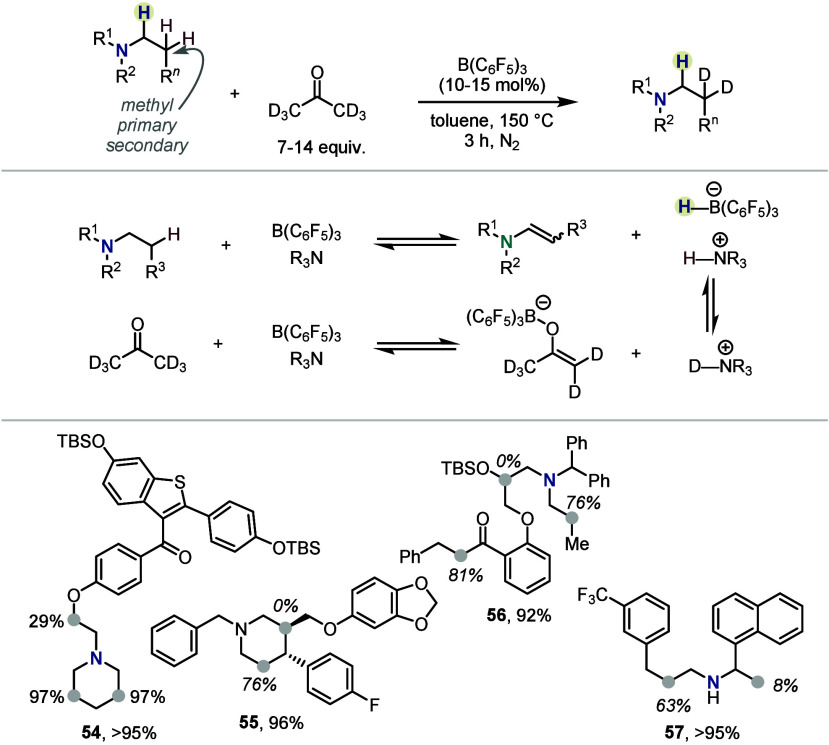
β-Deuteration of Amines by Wasa *et al.*

Wasa and co-workers then utilized this catalytic manifold for an isotope exchange reaction using deuterons as electrophiles and relying on reversible enamine formation.^45^ Thus, employing catalytic amounts of B(C_6_F_5_)_3_, the authors developed a protocol for the selective deuteration of the β-position of amines (Scheme 8). Deuterated acetone, shown to exchange its deuterium atoms for hydrogen atoms under the reaction conditions, was used as a readily available deuterium source. A wide range of cyclic and acyclic amines were selectively labeled in the β-position, with the level of deuteration consistently correlating with the hydridic character of the adjacent hydrogen or its steric availability. Thus, cyclic amines generally preferred exchange of the endo hydrogen atoms (**54**, Scheme 8), whereas branched substituents usually did not exchange (*e.g.*, position with 0% deuteration in **55**). Notably, also hydrogens α to carbonyl functionalities were exchanged in several cases (*e.g.*, 81% deuteration adjacent to the arylketone in **56**). Surprisingly, even a secondary amine **57** was found to be a suitable substrate and underwent successful deuteration instead of potentially inhibiting the catalyst. In this case, labeling of the β-position was also observed for the branched position, albeit in low amount (8%).

**Scheme 9:**
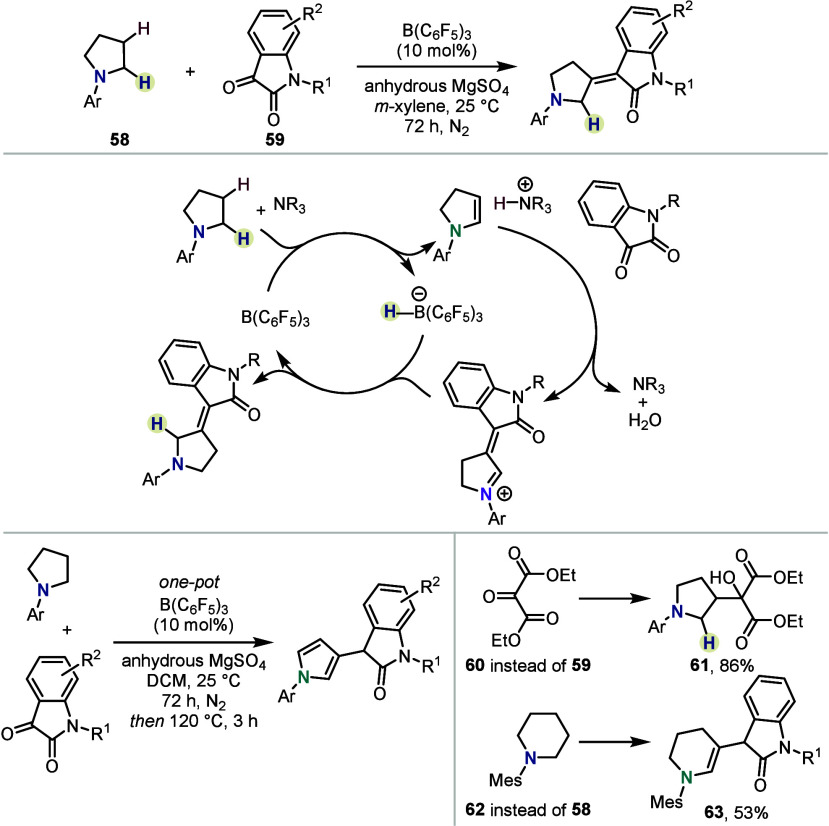
β-Functionalization with Isatins by Yang, Ma, *et al.*

Expanding the scope of suitable electrophiles, the groups of Yang and Ma introduced isatin derivatives, which could be efficiently coupled to cyclic aniline derivatives at room temperature (Scheme 9).^46^ While several solvents proved suitable, *m*-xylene was found to afford the highest stereoselectivity, yielding the alkene products with *Z*/*E* ratios between 4:1 and >20:1. Notably, while the initial reaction was performed at ambient temperature, the authors found that subsequent heating led to the formation of the corresponding pyrroles in high yields through acceptorless dehydrogenation. Interestingly, vicinal tricarbonyl **60** was also found to be a suitable electrophile for an enamine generated under these conditions—in contrast to other cases, however, the reaction proceeded without ultimate condensation. Changing from a pyrrolidine to a piperidine core led to desired product **63** with a migrated endocyclic double bond.

**Scheme 10:**
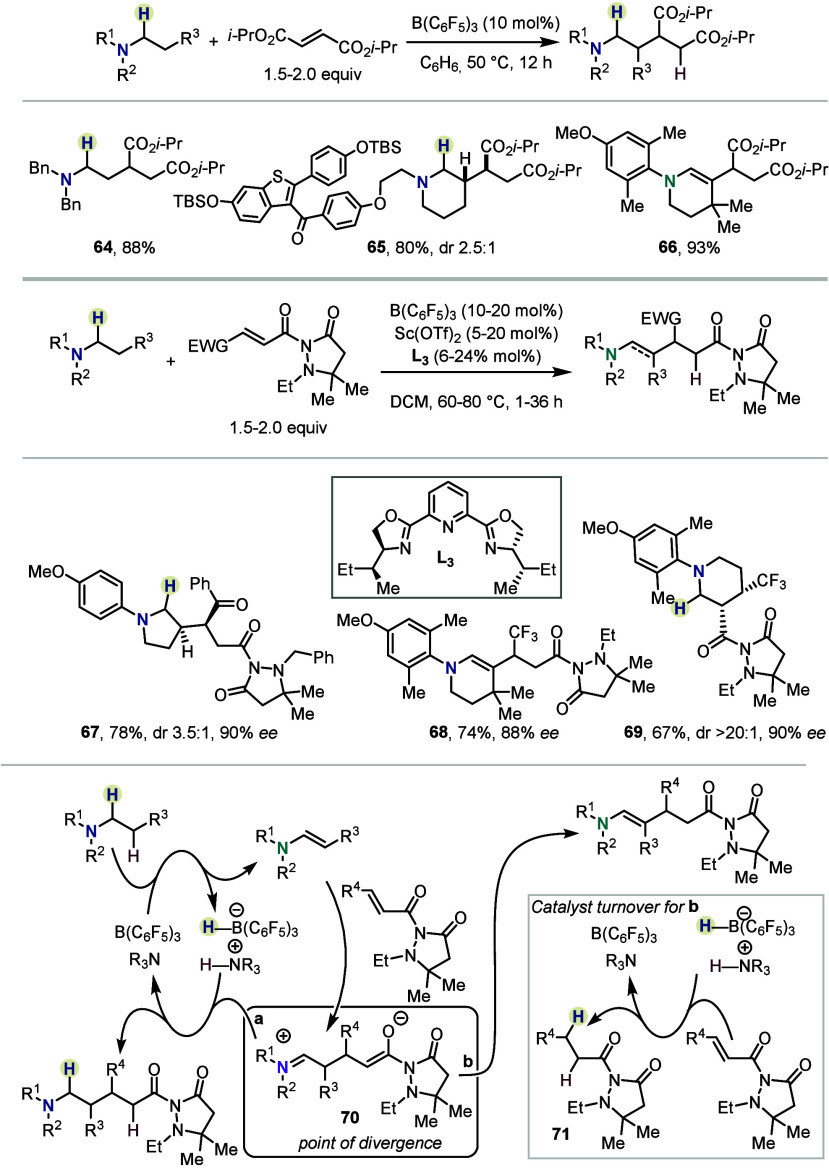
Enantioselective β-Functionalization by Wasa *et al.*

The β-functionalization of alkylamines with Michael acceptors, reported by Wasa and co-workers, marks a significant synthetic advancement (Scheme 10).^47^ There, divergent reactivity was observed depending on the substrates, leading either to the saturated addition products or the respective enamines. Interestingly, the selectivity of β- over α-functionalization of their previous work (see Scheme 1) seems to be dependent on the nature of the Michael acceptor, with acceptors featuring two electron-withdrawing groups favoring β-functionalization.

In addition to the racemic reaction, the transformation was rendered enantioselective by using acyl pyrazolidinone derivatives as electrophiles in combination with a scandium-based Lewis acid catalyst bearing a chiral PyBOX-based ligand **L**_**3**_. A wide range of anilines was coupled with good diastereomeric ratios and high levels of enantiomeric excess, again either forming the corresponding amines (*e.g.*, **67**) or enamines (*e.g.*, **68**). An interesting cyclization took place with ethyl methyl aniline which afforded **69** with excellent diastereo- and enantioselectivity. Mechanistically, the reaction’s divergent nature stems from intermediate **70** that can either undergo reduction to the amine (path a) or deprotonation to an enamine (path b). In the latter case, turnover is achieved by consumption of another equivalent of Michael acceptor to afford saturated acyl pyrazolidinones **71**.

**Scheme 11:**
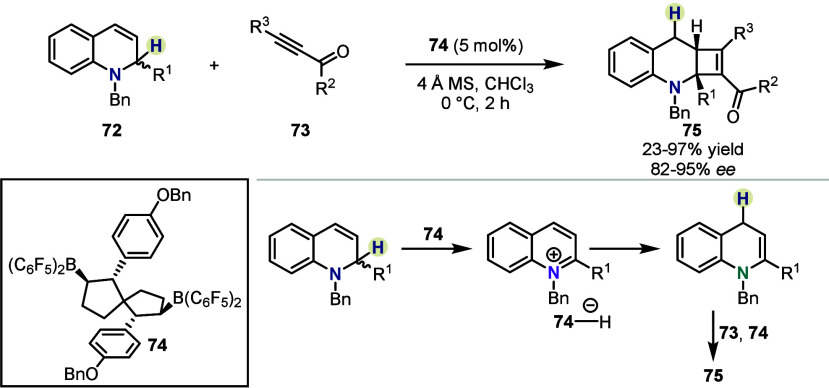
Isomerization and [2 + 2]-Cycloaddition by Wang *et al.*

Not only purely aliphatic amines, but also allyl amines can be used as precursors to form enamines *in situ*. Wang *et al.* demonstrated that 1,2-dihydroquinolines **72** can be isomerized to their corresponding enamines, capable of undergoing a formal [2 + 2]-cycloaddition with ynones **73** (Scheme 11).^48^ While B(C_6_F_5_)_3_ only afforded the product in low yield, the use of the dimeric chiral catalyst **74** led to high yields and enantioselectivities.

**Scheme 12:**
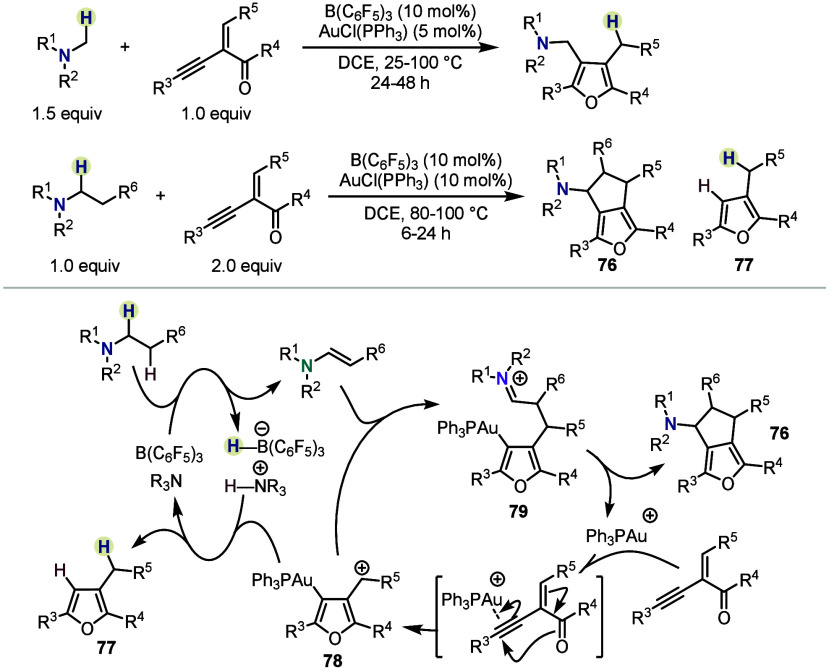
Borane/Gold(I)-Catalyzed C–H Functionalizations by Wang *et al.*

A further appealing avenue for alkylamine functionalization by the transient formation of enamines is the combination of hydride shuttle catalysis with other catalytic manifolds, such as gold catalysis. In 2022, Wang *et al.* reported a transformation enabling the synthesis of amine-functionalized furans from α-alkynyl enones, albeit without invoking intermediate enamines (Scheme 12, top).^49^ Notably, however, the authors also found that moving from methylamines to higher homologues triggered the formation of annulated products such as **76**. The proposed mechanism, on the one hand, involves enamine formation by the action of a Lewis acid catalyst and another amine molecule. Simultaneously, a gold-catalyzed furan synthesis forms an electrophilic intermediate (**78**), which reacts with the enamine. The thus formed intermediate **79** undergoes cyclization to yield the product **76**. Importantly, regeneration of the borane catalyst is achieved by the consumption of a second equivalent of ketone substrate, leading to byproduct **77**.

**Scheme 13:**
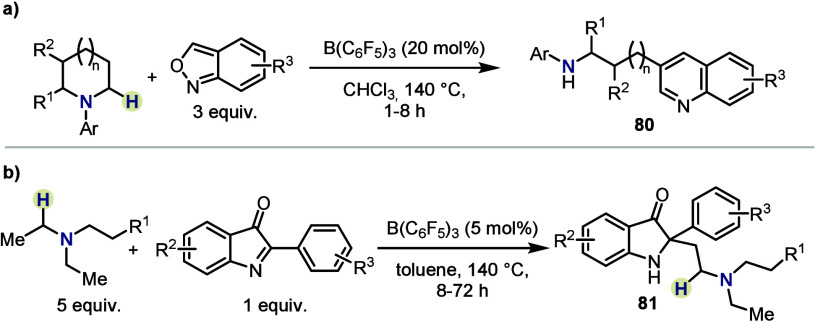
β-Functionalizations by (a) He, Fan *et al.* and (b) He, Zhao *et al.*

In recent years, the scope of suitable electrophiles was expanded toward cycloadditions with anthranil derivatives and addition to electrophilic imines, respectively (Scheme 13). The former transformation, reported by He, Fan and co-workers, led to functionalized quinolines. With cyclic amines, rearomatization after cycloaddition by elimination of the amine moiety led to amino-tethered products **80** (Scheme 13a).^50^

He and Zhao reported a related reaction that once more relies on high temperatures to overcome unproductive Lewis adduct formation in favor of the addition product, to afford β-functionalized amines **81** (Scheme 13b).^51^

**Scheme 14:**
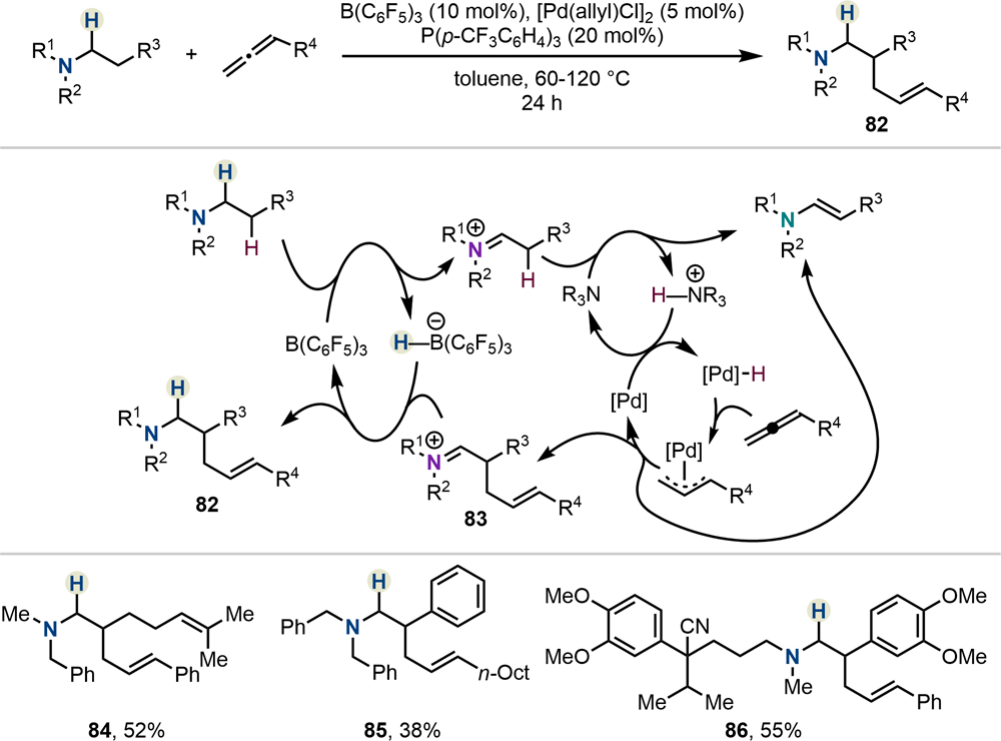
β-C–H Allylation of Trialkylamines by Wang *et al.*

With their synergistic use in combination with palladium-catalyzed reactions, borane-catalyzed hydride shuttle reactions have managed to jump yet another hurdle toward wide applicability in the synthetic toolbox of organic chemists. One such example has been presented by the Wang group in their synthesis of δ,ε-unsaturated amines **82** (Scheme 14).^52^ This intriguing reaction manifold utilizes allenes as coupling partners, following their *in situ* conversion to the corresponding electrophilic allyl palladium species. Addition of the *in situ*-generated enamine affords iminium intermediate **83** which is reduced to form the product with simultaneous regeneration of the borane catalyst. Both aromatic and aliphatic allenes were amenable to the reaction (**84**, **85**). Product **86** revealed the benzylic position to be allylated preferentially, likely due to the preferential formation of the corresponding styrene intermediate.

**Scheme 15:**
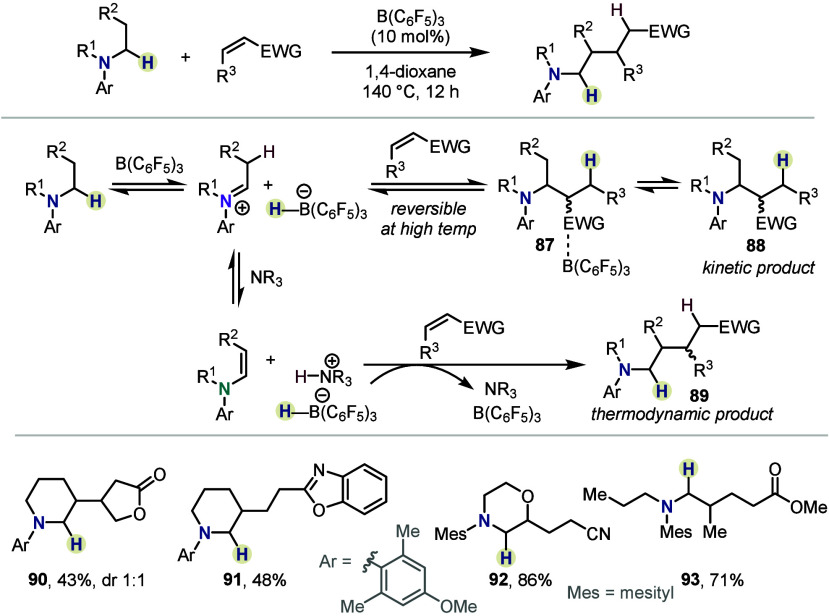
β-C–H Functionalizations with Michael Acceptors by Xue, Wang *et al.*

Most recently, the Wang group extended β-functionalization reactions of amines to Michael acceptors (Scheme 15).^53^ While these were known to afford α-functionalization (*cf.*Scheme 1), the authors demonstrated that, at high temperature, the Mannich addition toward **88** becomes reversible, allowing the reaction to funnel into the thermodynamic product **89**. Under the reaction conditions, β,β-difunctionalized amines were also observed as byproducts, adding two Michael acceptors to both alkyl substituents (not shown). Several cyclic Michael acceptors (*cf.***90**), as well as vinyl heteroarenes (*cf.***91**), were successfully coupled to anilines bearing cyclic or acyclic alkyl substituents (*cf.***92**, **93**).

These examples show that the β-functionalization of amines by virtue of hydride shuttle catalysis has constituted a breakthrough in synthetic utilization and especially the prospect of cooperative catalysis.

## C Inverse Hydride Shuttle Catalysis

**Scheme 16:**
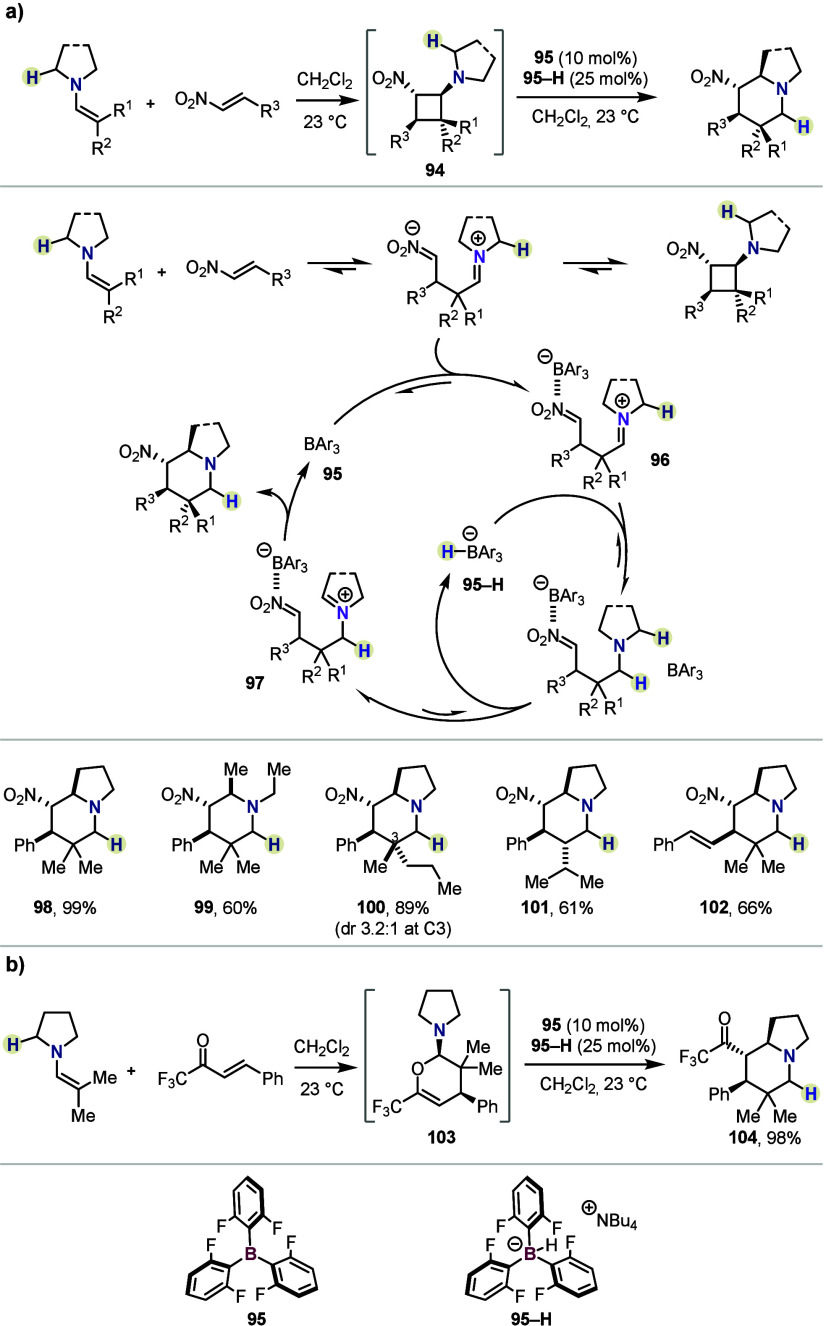
Inverse Hydride Shuttle Catalysis by Maulide *et al.*

HSC transformations are triggered by initial α-hydride abstraction from an amine by a triarylborane catalyst. A conceptually related, yet mechanistically distinct, approach was developed by the Maulide group in 2022, enabling the multicomponent conversion of amines, aldehydes and Michael acceptors (*e.g.*, nitrostyrenes) to valuable azabicyclic scaffolds with exquisite diastereoselectivity (Scheme 16).^54^ This process was termed “inverse hydride shuttle catalysis” (*i*-HSC), as the key event is triggered by *delivery* of a hydride, rather than abstraction (Figure 1C). Dynamically assembled ternary complexes, such as cyclobutane **94**, undergo a skeletal rearrangement upon exposure to a unique catalyst mixture (consisting of Lewis acid **95** and its hydride **95–H**). Transient cyclobutane **94** is believed to be in equilibrium with its open zwitterionic form **96**, where successive reduction of the iminium moiety, followed by hydride abstraction at the most sterically accessible position, provides intermediate **97** prior to ring closure. A range of azabicyclic products was accessed in high yield and as single diastereomers bearing three contiguous stereocenters (*e.g.*, **98**–**102**). An attractive feature of *i*-HSC is the potential telescoping of the process to include initial enamine formation from aldehydes and secondary amines *in situ*, in comparable yield (not shown). Furthermore, in addition to nitrostyrenes, trifluoromethyl enones were also found to be suitable substrates (Scheme 16b). Here, aminodihydropyran **103** is the observable resting state following Michael addition, rather than the cyclobutane. It undergoes an analogous rearrangement to give products of type **104** in excellent yield and diastereoselectivity.

**Scheme 17:**
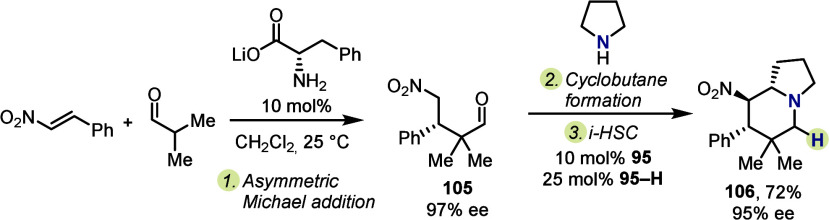
Enantioselective Inverse Hydride Shuttle Catalysis

Notably, changing the order of events in the previously described protocol allows for an enantioselective approach (Scheme 17). A separate, organocatalytic asymmetric Michael addition provided aldehyde **105** with high enantiomeric excess, at which point cyclobutane formation (triggered by addition of pyrrolidine) and catalytic *i*-HSC provided **106** in high yield and without loss of enantioenrichment. It is important to highlight that this particular process is limited to nitrostyrenes, as the presence of the nitro group allows for *in situ* deprotonation, thereby enabling closure to the enantioenriched cyclobutane from aldehyde **105**.

**Scheme 18:**
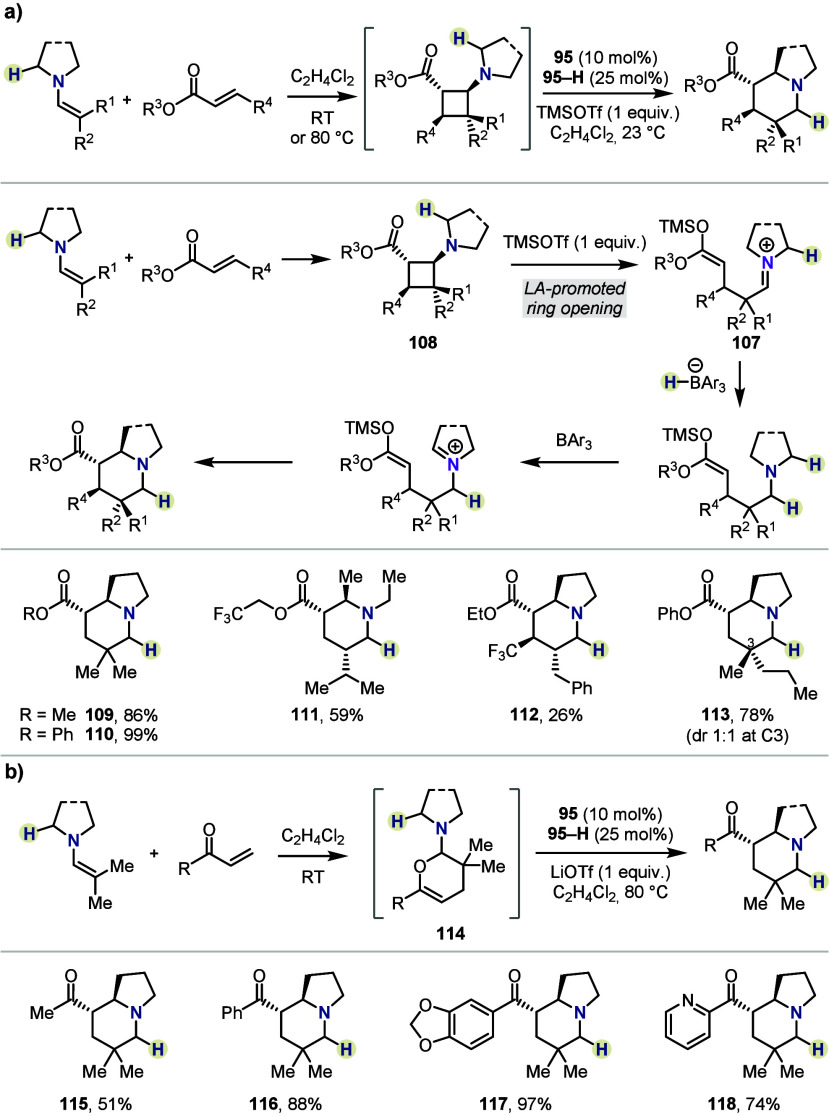
Lewis Acid-Driven Inverse Hydride Shuttle Catalysis by Maulide *et al.*

An important limitation of the first generation of *i*-HSC was the requirement for highly electron-withdrawing and non-Lewis basic Michael acceptors to facilitate the ring opening of transient cyclobutane (*e.g.*, **94**) or aminodihydropyran (*e.g.*, **103**) intermediates. Intermediates derived from less electron-deficient acceptors, such as acrylates, exhibit resistance to ring opening, preventing their use in *i*-HSC. Subsequent work from the Maulide group addressed this constraint through the introduction of a complementary Lewis acid to facilitate the ring opening of such intermediates, and significantly expanded the scope of accessible products (Scheme 18a).^55^ Addition of trimethylsilyl triflate (TMSOTf) to the second operation promoted the formation of silyl ketene acetal/iminium species **107** from cyclobutane **108**. Subsequent iminium isomerization through *i*-HSC, followed by ring closure, gave a range of ester-substituted azabicycles with varied substitution patterns (*e.g.*, **109**–**113**). Additionally, using the same approach, unsubstituted enones were also rendered suitable substrates, where LiOTf was an effective promoter for the ring opening of aminodihydropyrans of type **114**, providing both alkyl (*e.g.*, **115**) and aryl (*e.g.*, **116**–**118**) ketone-substituted products as single diastereomers (Scheme 18b).

**Scheme 19:**
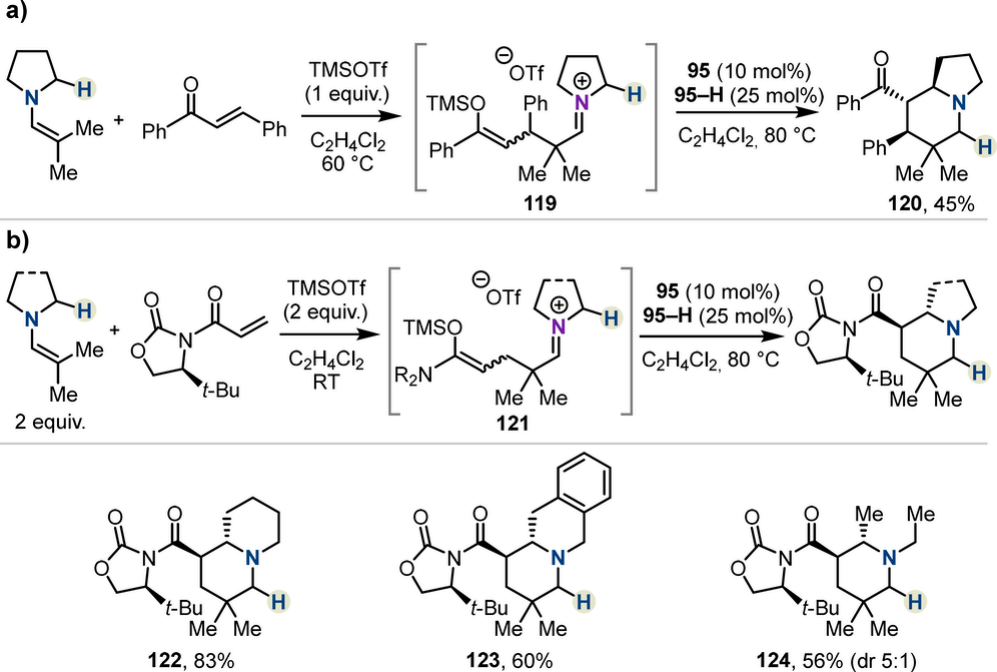
Alternative Strategy for Lewis Acid-Driven Inverse Hydride Shuttle Catalysis by Maulide *et al.*

An alternative strategy to encompass acceptors for which initial Michael addition is challenging is outlined in Scheme 19a. Here, TMSOTf was added to the first operation to promote the Michael addition between an enamine and a substituted enone. The observable resting state intermediate in this case is linear silyl enol ether/iminium ion **119**, which readily undergoes *i*-HSC and ring closure to generate products of type **120** in modest yield. Finally, a streamlined asymmetric cyclization was achieved using a chiral oxazolidinone on the acceptor fragment (Scheme 19b). Addition of two equivalents of TMSOTf allowed for full conversion to linear iminium intermediate **121** en route to enantioenriched products (**122**–**124**) with excellent diastereoselectivity. Notably, **123** was formed as a single regioisomer, a consequence of selective hydride abstraction at the most sterically accessible position. This protocol allows streamlined access to enantioenriched products with enhanced opportunities for downstream modifications, ultimately facilitating the application of *i*-HSC to the synthesis of interesting alkaloids. This synthetic advantage was presented in a concise total of (−)-tashiromine.

## D Conclusions and Outlook

In this perspective, we have aimed to provide an overview of distinct modes of hydride shuttle catalysis, which leverage the unique interaction of hindered amines with a triarylborane catalyst. Sterically congested donor–acceptor partners of this type cannot form a conventional Lewis pair, and instead form an iminium-borohydride ion pair following α-hydride abstraction from the amine component. Although this chemistry is largely underrepresented in the literature, a variety of powerful methods have been developed, encompassing either direct trapping of the initial iminium species, or subsequent isomerization to an enamine. Collectively, these approaches provide highly efficient routes to α- and β-functionalized products, formed with high levels of stereocontrol. More recently, inverse hydride shuttle catalysis has been introduced as a highly efficient strategy to access diverse azabicyclic molecules. These processes are triggered by hydride delivery, rather than abstraction, and proceed with full diastereocontrol. Several elegant enantioselective transformations which exploit both conventional and inverse modes have emerged, showcasing the potential of hydride shuttle catalysis. We anticipate this actively evolving area of research to receive increasing attention from the synthetic community in the coming years.
